# The emergency department landscape in The Netherlands: an exploration of characteristics and hypothesized relationships

**DOI:** 10.1186/s12245-018-0196-5

**Published:** 2018-09-05

**Authors:** Menno I. Gaakeer, Rebekka Veugelers, Joris M. van Lieshout, Peter Patka, Robbert Huijsman

**Affiliations:** 1 Department of Emergency Medicine, Admiraal De Ruyter Hospital, Goes, The Netherlands; 2 Department of Emergency Medicine, Erasmus University Medical Center, Rotterdam, The Netherlands; 3 Erasmus School of Health Policy & Management, Erasmus University Rotterdam, Rotterdam, The Netherlands

## Abstract

**Background:**

Nationwide optimization of the emergency department (ED) landscape is being discussed in The Netherlands. The emphasis is put mostly on the number of EDs actually present at the time versus a proposed minimum number of EDs needed in the future. The predominant idea in general is that by concentrating emergency care in less EDs costs would be saved and quality of care would increase. However, structural insight into similarities as well as differences of ED characteristics is missing. This knowledge and fact interpretation is needed to provide better steering information which could contribute to strategies aiming to optimize the ED landscape. This study provides an in-depth insight in the ED landscape of The Netherlands by presentation of providing an overview of the variation in ED characteristics and by exploring associations between ED volume characteristics on one side and measures of available ED and hospital resources on the other side. Obtained insight can be a starting point towards a more well-founded future optimization policy.

**Methods:**

This is a nationwide cross-sectional observational study. All 24/7 operational EDs meeting the IFEM definition in The Netherlands in December 2016 were identified, contacted and surveyed. Requested information was retrieved from local hospital information systems and entered into a database. Till August 1, 2017, data have been collected.

**Results:**

All 87 eligible EDs in The Netherlands participated in this study (100%). All of them were hospital based. These were 8 EDs in universities (9%), 27 EDs in teaching hospitals (31%) and 52 EDs in general hospitals (60%). On average, 22,755 patients were seen per ED (range 6082–53,196). On average, 85% (range 44–99%) was referred versus 15% self-referred (range 1–56%). Further subdivision of the referred patients showed 17% ‘emergency call’ (range 0.5–30%), 52% by GPC (range 16–77%) and 15% other referral (range 1–52%). On average, 38% of patients per ED (range 13–76%) were hospitalized. ED treatment bays ranged from 4 to 36 and added nationally up to 1401 (mean and median of 16 per ED). The number of hospital beds behind these EDs ranged from 104 to 1339 and added up to 36,630 beds nationally (mean of 421 and median of 375 behind each ED). Information about ED nurse workforce was available for 83 of 87 EDs and ranged from 11 to 65, adding up to 2348 fulltime-equivalent nationally (mean of 28 and median of 27 per ED).

We found positive and significant correlations, confirming all formulated hypotheses. The strongest correlation was seen between the number of patients seen in the ED and ED nurse workforce, followed by the number of patients seen in the ED and ED treatment bays. The other hypotheses showed less positive significant correlations.

**Conclusion:**

Our study shows that the ED landscape is still pluriform by numbers and specifications of individual ED locations. This study identifies associations between patient and hospitalization volumes on a national level on one side and number of ED treatment bays, ED nurse workforce capacity and available hospital beds on the other side. These findings might be useful as input for the development of an ED resource allocation framework and a more targeted optimization policy in the future.

## Background

Nationwide optimization of the emergency department (ED) landscape is being discussed in many countries all over the world for reasons such as cost reduction and quality improvement. In The Netherlands, one such country, several guiding reports on this topic have been published [1–3]. The emphasis here was mostly on the number of EDs actually present at the time versus a proposed minimum number of EDs needed in the future at which sufficient ED accessibility to the population could be maintained [1–3]. The predominant idea was that by concentrating emergency care in fewer EDs costs would be saved and quality of care would increase. The 45-min standard (45-min time limit for ambulance services to deliver patients to an ED, including arriving at the patient within 15 min after being dispatched) applies as a restrictive measure for this sufficient accessibility from the Dutch government [4, 5]. In line with the development of last decades, the number of EDs has declined gradually from 105 in 2010 to 87 in 2016. In all cases, that was a result of mergers of hospital organizations [6]. However, whether the intended nationwide optimization goals of cost reduction and quality improvement are achieved is being questioned [7–10]. At the same time, pressure across the width of the acute care system has increased and in particular crowding in EDs with all its negative aspects has become a growing problem [11–13]. We assumed that reducing the number of EDs as strategy in itself has limited effectiveness. This might be among other things because in practice a broad variation of departments in many ways is hiding behind the commonly used term ED. The diversity within the ED landscape in The Netherlands has been demonstrated earlier [4, 6, 14]. However, structural insight into similarities as well as differences of ED characteristics is missing. This knowledge is needed to provide better steering information which will contribute to strategies aiming to optimize the ED landscape.

With this study, we aimed to provide additional insight in the ED landscape of The Netherlands in two ways. First is by providing a national overview of the variation in ED characteristics. Secondly, we investigated the association between ED volume characteristics (number of patients and hospitalizations) on one side and measures of available ED and hospital resources (ED treatment bays, ED nursing staff and number of hospital beds) on the other side. Obtained insight can be a starting point towards more well-founded future optimization policy.

## Methods

### Design

This is a nationwide cross-sectional observational study of administrative data. All 24/7 available and operational EDs meeting the IFEM definition in The Netherlands in December 2016 were identified, contacted and surveyed. In January 2017, a questionnaire was emailed, asking index contacts about key administrative data (reference date December 31, 2016). Requested information was retrieved from local hospital information systems and sent by email to the first author, who entered all information in a database. Till August 1, 2017, data have been collected. Over this period, every month a prompt was given by email to those who had not responded yet. Our goal beforehand was to obtain participation of at least of 80% of all eligible EDs. Participation was entirely voluntary and withdrawal from the study could occur at any point.

### Setting

The Netherlands is a West-European country with 17 million inhabitants and is one of the most densely populated countries worldwide (500 people per km^2^ if water is excluded). Its gross domestic product (GDP) in 2016 was 770.85 billion of US Dollar and total expenditure on health care in that year was 13.6% of GDP [15, 16]. In 2016 life expectancy at birth for both sexes was estimated at 79.9 years for males and 83.1 years for females [17]. Primary care is highly developed and accessible to everyone through local general practitioner (GP) offices during daytime and GP cooperatives during the evenings, nights and weekends. GPs fulfill the role of gatekeepers for hospital care. At the end of 2016, emergency care was provided in 87 EDs with 24/7 availability spread over the country [6]. To date, there are over 540 trained and registered emergency physicians (EPs) working in 85% of all EDs. Annually about 115–124 ED presentations per 1000 inhabitants were recorded [6]. Almost all citizens have health care insurance, but with a mandatory excess (in Dutch: verplicht eigen risico) for ED/hospital-based care.

### Eligible EDs

Worldwide, the term ED is being used to refer to a broad range of urgent care facilities. The International Federation for Emergency Medicine (IFEM) terminology project defined the entity of an ED in 2012 as: ‘The area of a medical facility devoted to provision of an organized system of emergency medical care that is staffed by Emergency Medicine Specialist Physicians and/or Emergency Physicians (EPs) and has the basic resources to resuscitate, diagnose and treat patients with medical emergencies. The ED is a unique location at which patients are guaranteed access to emergency care 24 hours a day, 7 days a week. It is able to deal with all types of medical emergencies (illness, injury and mental health) in all age groups’ [18]. To be included in our study, EDs needed to meet all the following criteria from the IFEM definition in December 2016:

- Centrally organized location for the reception of patients in need for emergency care,
- Around the clock (24/7) availability and accessibility,
- Being able to deal with illness, injury and mental health emergencies,
- Serving all age categories.

### ED characterization

There is no (inter)nationally validated instrument to profile EDs. In previous conducted research, ED characterization has been done in different ways, all with their own added value [19–26]. Based on literature and guided by both our study aims, including pre-formulated hypotheses, we consciously focused on the following administrative data. *Patient characteristics*: volume of visits (patients per year); distribution of age (0–18, 18–65, 65+); route of presentation: referred (‘emergency call’; by GP(C); other referral) versus self-referral; hospital admissions through the ED (patients per year). *ED and hospital capacity*: number of ED treatment bays; number of hospital beds. *Workforce capacity*: fulltime-equivalent (fte) of ED nurses. In addition, the following information with respect to each ED was gathered:

- Method of cooperation with a general practitioner cooperative (GPC):
- Co-location: ED and GPC are located separately.
- Parallel: a GPC is located at a hospital and has its own reception desk within the ED. Separate triage procedure.
- Serial: a GPC is located at the hospital, with a reception desk earlier in line than the hospital’s. Serially, the GPC is thus positioned before the ED. Separate triage procedure.
- Integrated: the GPC and ED share a reception desk. Common triage procedure.
- Category of hospital the ED is located in (university; STZ; general). STZ is an exclusive partnership of 26 non-university training hospitals throughout The Netherlands. Together these hospitals house 27 EDs.

### Hypothesized relationships

In contrast to intensive care units, coronary care units and operating rooms, a national framework with binding recommendations for ED resource planning is not available in The Netherlands. This seems primarily a local responsibility of hospital organizations. However, ED volumes are assumed to be relevant parameters for ED resource planning in several ways [27–31]. Despite the lack of a framework, we wondered whether a number of obvious relationships between volumes on one side and resources on the other side actually could be demonstrated in the ED landscape of The Netherlands. In order to study these relationships, the authors pre-formulated six hypotheses listed below, which we aimed to confirm or refute.

ED patient volume versus available resources:

- *Hypothesis 1*: ED patient volumes correlate positively with numbers of ED treatment bays.
- *Hypothesis 2*: ED patient volumes correlate positively with numbers of hospital beds.
- *Hypothesis 3*: ED patient volumes correlate positively with volumes of ED nurse staffing.

Hospitalizations through the ED versus available resources:

- *Hypothesis 4*: Hospitalization volumes correlate positively with numbers of ED treatment bays.
- *Hypothesis 5*: Hospitalization volumes correlate positively with numbers of hospital beds.
- *Hypothesis 6*: Hospitalization volumes correlate positively with volumes of ED nurse staffing.

### Data analysis

Our analysis included two steps. First, we used descriptive statistics to present current baseline ED landscape characteristics and the variation thereof. Second, to confirm or refute the hypotheses, we used the Pearson correlation coefficient and calculated the adjusted *R*^2^. Significance threshold was set at a *p* value of 0.05.

### Ethics

Ethical approval for this study was obtained from the Medical Research Ethics Committee of the Erasmus University Medical Center in Rotterdam, The Netherlands (Ref. MEC-2014-322).

## Results

### Eligible EDs and participation

All 87 eligible EDs in The Netherlands participated in this study (100%).

### Characteristics of EDs

Every ED was hospital based. This were 8 university EDs (9%), 27 EDs in STZ hospitals (31%) and 52 in general hospitals (60%). Seventy-one EDs (82%) cooperated with a GPC located in the hospital. Of these, 21 GPCs were located parallel, 25 serial and 25 integrated with the ED. The percentage registered self-referrals on average was 13%, 8% and 21% respectively. Sixteen EDs (18%) cooperated with a GPC located outside the hospital with on average 19% self-referrals.

The profile of further ED characteristics is presented in Table 1. A total of 1,979,726 patients were seen in all EDs together in 2016. This corresponds with an average of 22,755 patients per ED (range 6082–53,196) and 116 patients per 1000 inhabitants. Of all patients, 18.2% (range 9–30%) was 0 up to 18 years, 48.4% (range 39–69%) was 18 up to 65 years and 33.8% (range 13–51%) was 65 years and older. On average, 85% (range 44–99%) was referred versus 15% self-referred (range 1–56%). Further subdivision of the referred patients showed 17% ‘emergency call’ (range 0.5–30%); 52% by GPC (range 16–77%); 15% other referral (range 1–52%). In total, 728.804 of all ED patients were admitted through all EDs together, on average 38% of patients per ED (range 13–76%). ED treatment bays ranged from 4 to 36 and added nationally up to 1401 (mean and median of 16 per ED). The number of hospital beds behind these EDs ranged from 104 to 1339 and added up to 36,630 beds nationally (mean of 421 and median of 375 behind each ED). Information about ED nurse workforce was available for 83 EDs and ranged from 11 to 65, adding up to 2348 fte nationally (mean of 28 and median of 27 per ED).

**Table 1:** ED characteristics

	Mean ± sd	Min	Max
Patient volume and characteristics			
ED patients (per year)	22,755 ± 9457	6082	53,196
Age patients			
% < 18	18.2 ± 4.5	9	30
% 18–64	48.4 ± 5.9	39	69
% 65+	33.8 ± 7.5	13	51
Entry route patients			
% self-referrals	14.9 ± 13.5	1.2	55.9
% primary care	51.8 ± 14.4	16.4	76.5
% EMS	16.6 ± 5.3	0.5	30.0
% other	15.2 ± 8.0	1.1	52.1
% admissions	38.2 ± 10.7	12.9	76.4
ED and hospital capacity			
ED beds	16.1 ± 6.6	4	36
Hospital beds	441.3 ± 245.3	104	1339
Workforce capacity			
FTE ED nurses	28.3 ± 11.0	11	65
Calculated ratios			
FTE ED nurse/ED bed	1.8 ± 0.5	0.9	3.5
FTE ED nurse/ED patient (per 24 h)	0.46 ± 0.97	0.30	0.72
Admissions/FTE ED nurse (per 24 h)	0.86 ± 0.31	0.18	2.24
ED beds/patient (per 24 h)	0.27 ± 0.08	0.16	0.54

### Hypothesized relationships

**Fig. 1:**
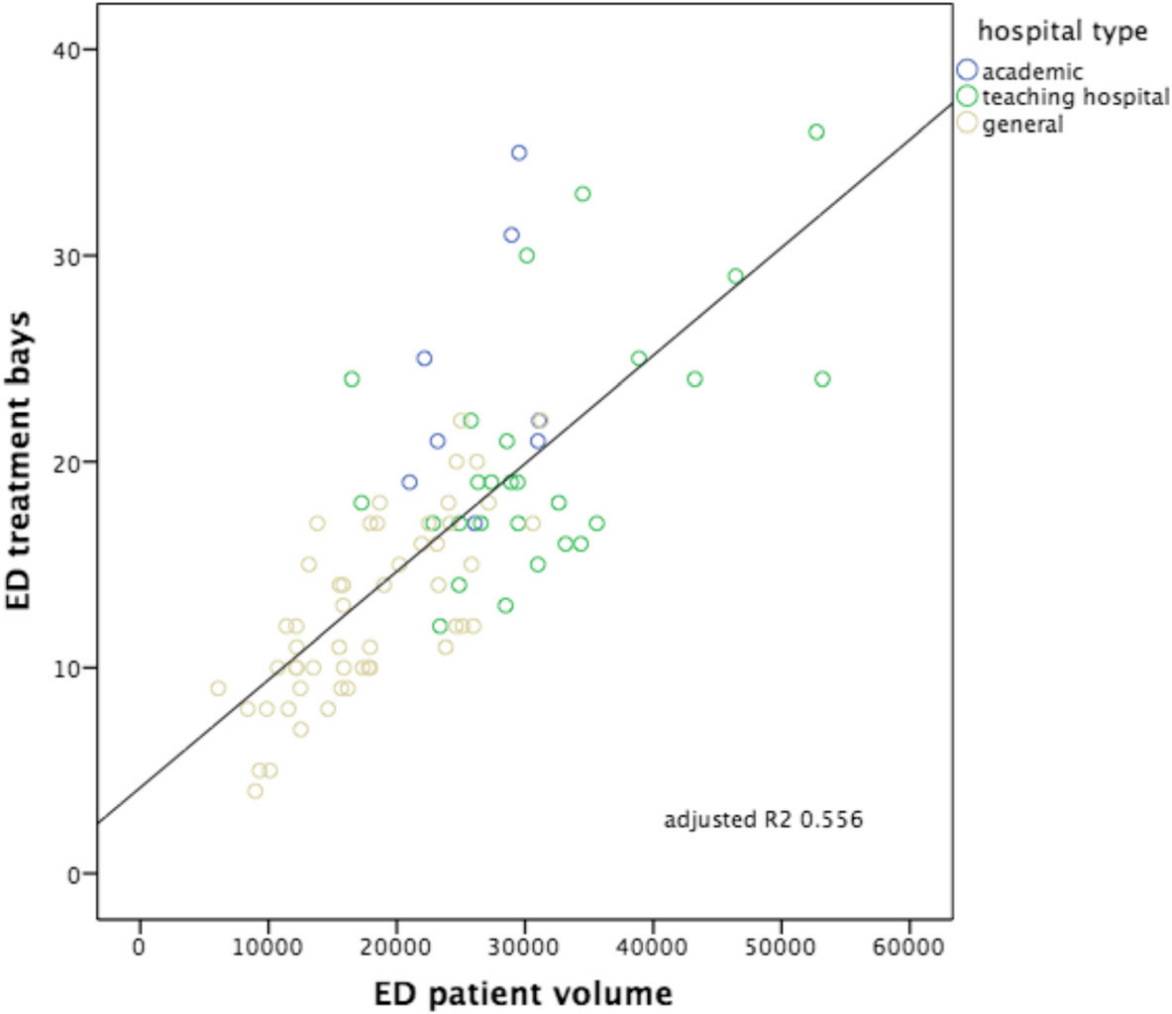
ED patient volumes versus numbers of ED treatment bays. Subdivision to hospital type: academic, teaching hospital and general hospital. Including correlation line with calculated adjusted *R*^2^

**Fig. 2:**
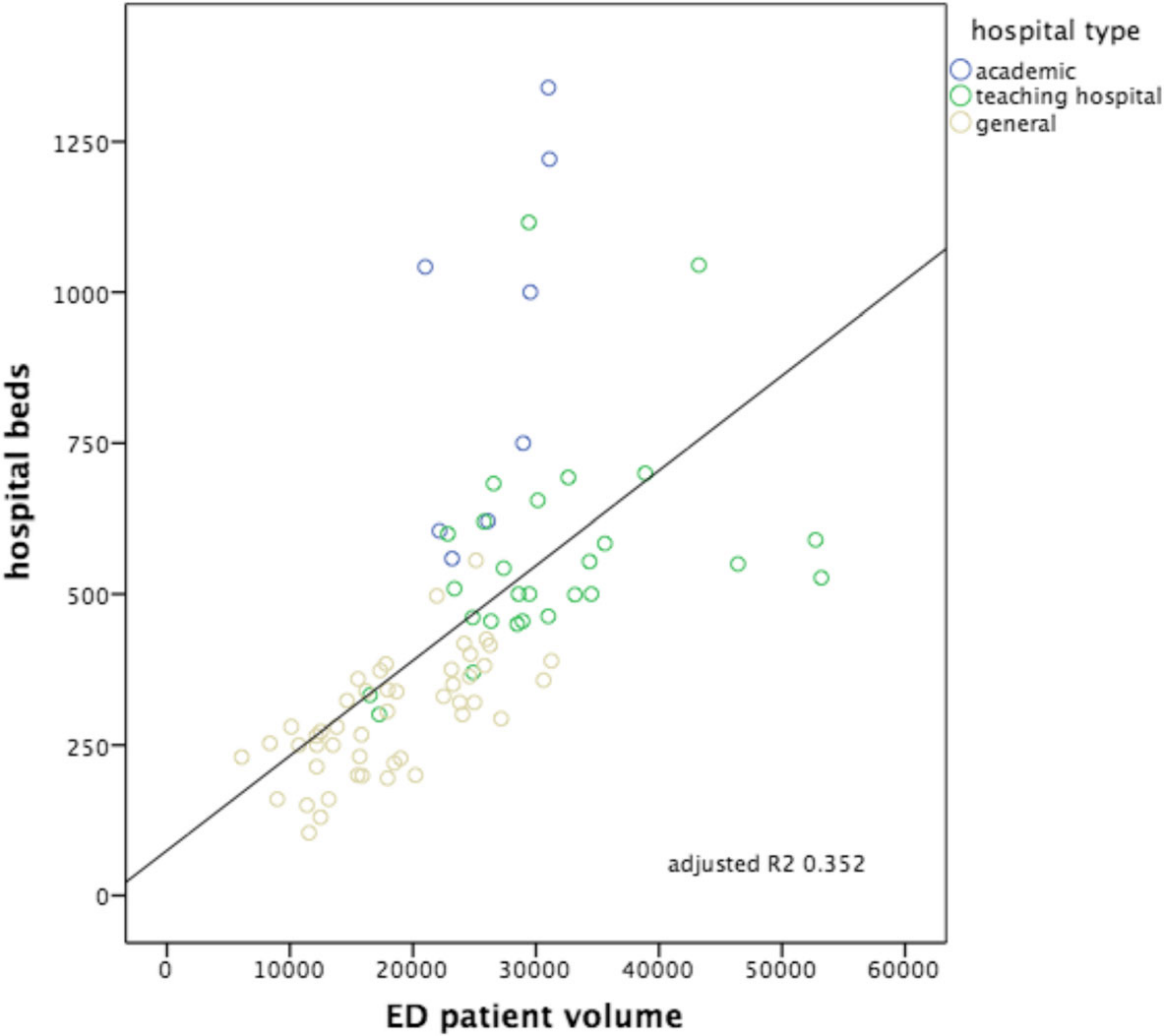
ED patient volumes versus numbers of hospital beds. Subdivision to hospital type: academic, teaching hospital and general hospital. Including correlation line with calculated adjusted *R*^2^

**Fig. 3:**
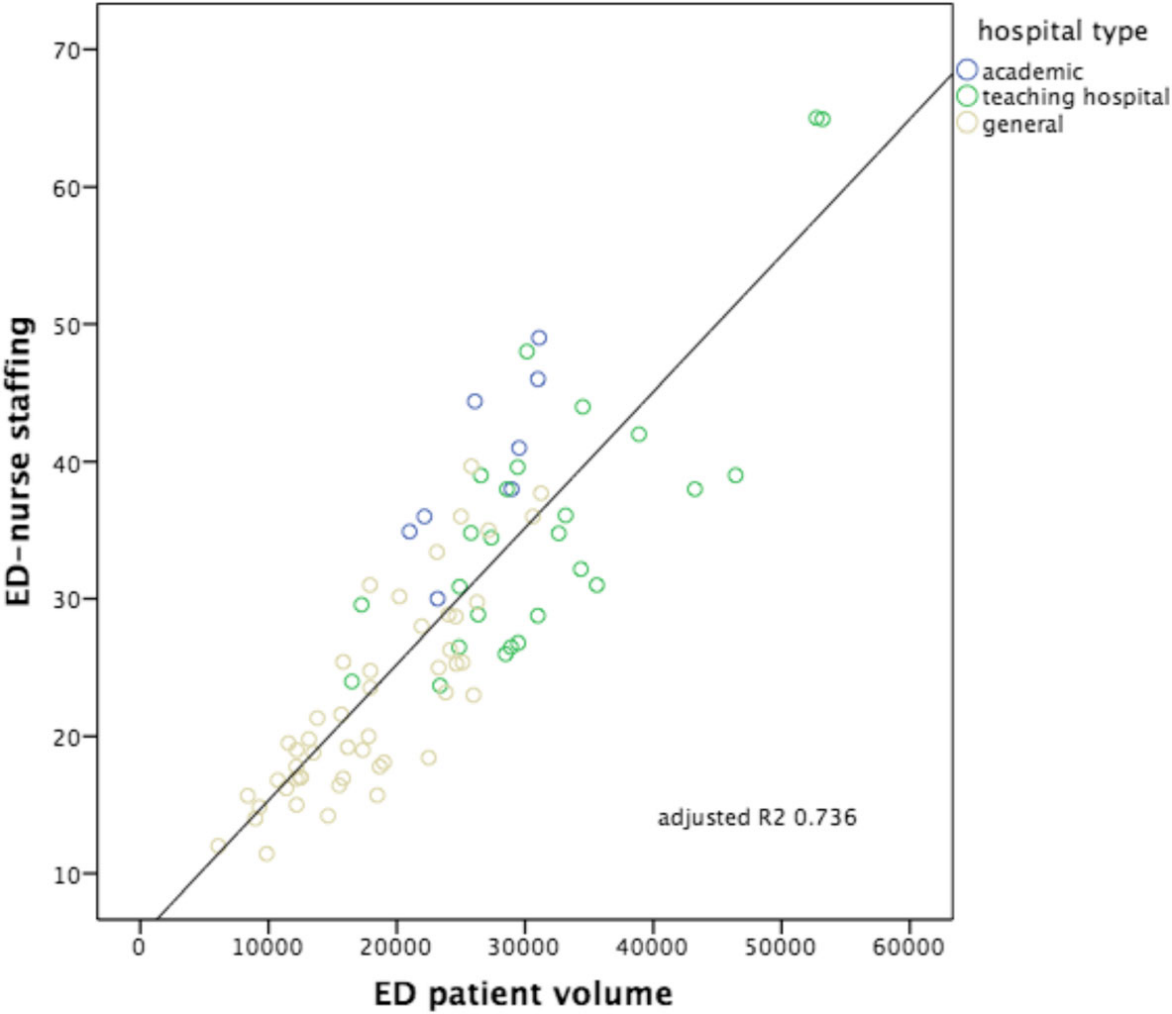
ED patient volumes versus volumes of ED nurse staffing. Subdivision to hospital type: academic, teaching hospital and general hospital. Including correlation line with calculated adjusted *R*^2^

**Fig. 4:**
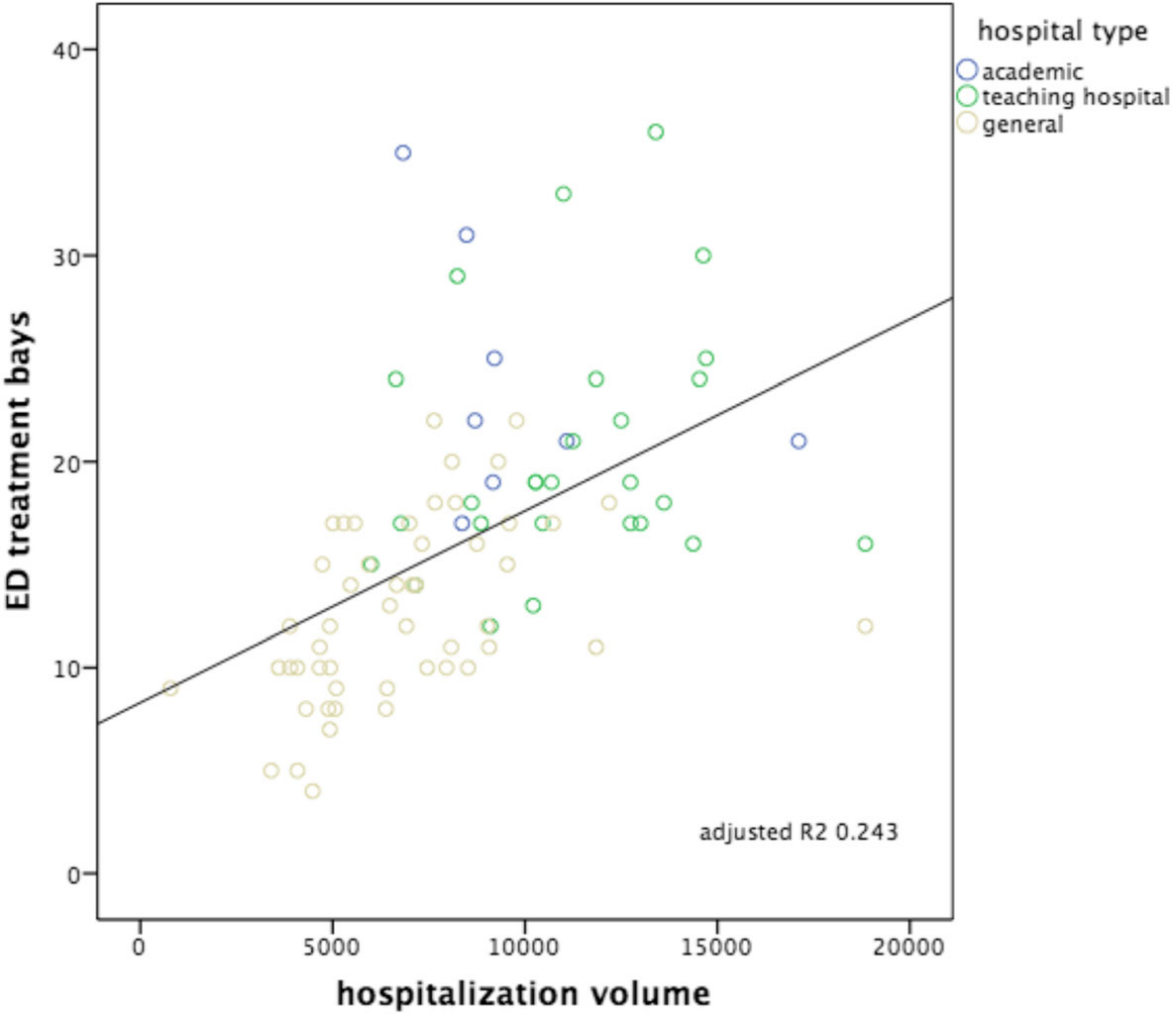
Hospitalization volumes versus numbers of ED treatment bays. Subdivision to hospital type: academic, teaching hospital and general hospital. Including correlation line with calculated adjusted *R*^2^

**Fig. 5:**
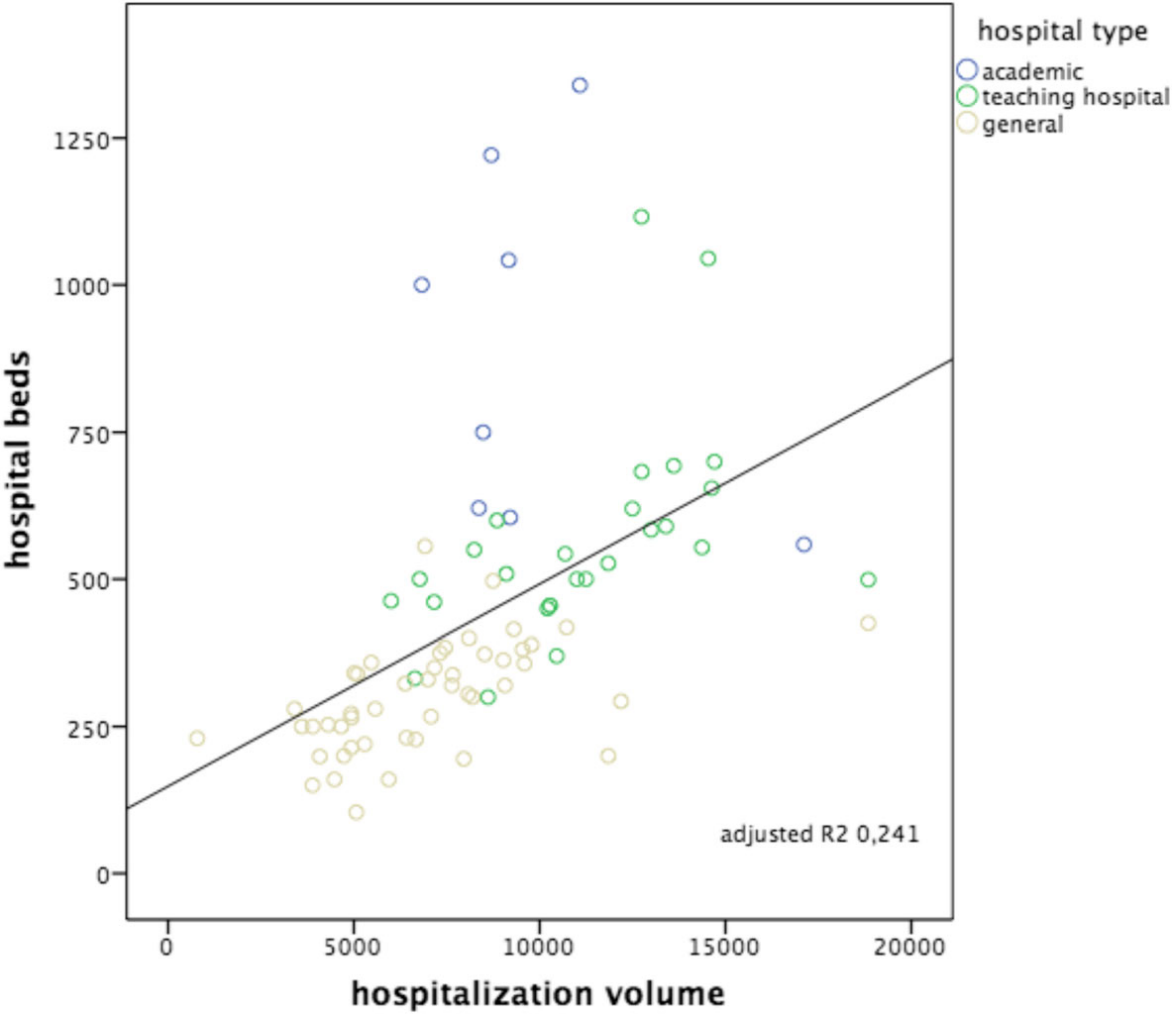
Hospitalization volumes versus numbers of hospital beds. Subdivision to hospital type: academic, teaching hospital and general hospital. Including correlation line with calculated adjusted *R*^2^

**Fig. 6:**
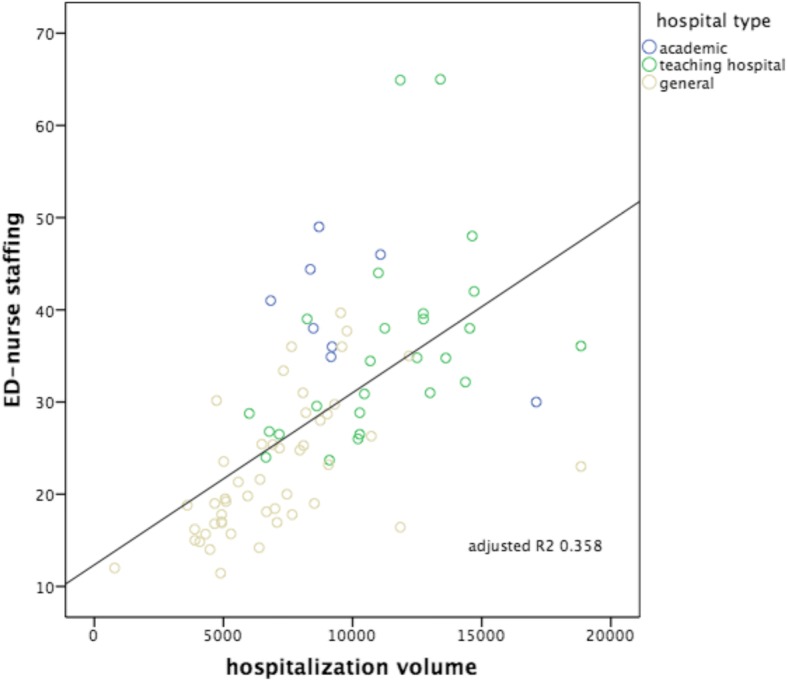
Hospitalization volumes versus volumes of ED nurse staffing. Subdivision to hospital type: academic, teaching hospital and general hospital. Including correlation line with calculated adjusted *R*^2^

The scatterplots with regression line and calculated adjusted *R*^2^ for each of the six hypotheses are shown in Figs. 1, 2, 3, 4, 5 and 6. We found positive and significant correlations, confirming all hypotheses.

The strongest correlation was seen between the number of patients seen in the ED versus ED nurse workforce (hypothesis 3: Adj *R*^2^: 0.736, *p* < 0.000), followed by the number of patients seen in the ED versus ED treatment bays (hypothesis 1: Adj *R*^2^: 0.556, *p* < 0.000). The other hypotheses showed smaller though significant correlations, in decreasing order: hospitalization volumes versus volumes of ED nurse staffing (hypothesis 6: Adj *R*^2^: 0.358, *p* < 0.000); ED patient volumes versus the numbers of hospital beds (hypothesis 2: Adj *R*^2^ 0.352, *p* < 0.000); hospitalization volumes versus the numbers of ED treatment bays (hypothesis 4: Adj *R*^2^: 0.243, *p* < 0.000); hospitalization volumes versus the numbers of hospital beds (hypothesis 5: Adj *R*^2^: 0.241, *p* < 0.000). Despite these correlations, all six scatterplots show large variation, also within each different subgroup of EDs (academic, teaching and general). All academic EDs are located almost consistently above the correlation lines, indicating more resources available in relation to patient volume or hospitalization rate in comparison with teaching or general EDs. An obvious explanation for this could be that they all have tertiary care functions and are level I trauma centers and therefore need more resources we examined. It is remarkable that although the academic EDs handle and hospitalize a comparable number of patients, the number of available resources between themselves varies enormously. General EDs are located almost consistently below the correlation lines, indicating more patients or hospitalizations with less resources. A valid insight into the differences in workload between different types of EDs as possible explanation for the variance in resources is lacking.

## Discussion

This study, to our knowledge the first in its kind, demonstrates that the ED landscape in The Netherlands consists of a broad variety of EDs in regard to their individual profiles. In addition, on a national level this study identifies associations between patient and hospitalization volumes on one side and number of ED treatment bays, ED nurse workforce capacity and available hospital beds on the other side. These findings form a first step towards more insight into the ED landscape present and might be useful as input for the development of an ED resource allocation framework and more targeted optimization policy in the future.

Based on characteristics, we examined EDs in The Netherlands apparently cannot be considered as equals. In line with findings of previous research, these departments continue to show a pluriform landscape [4, 6, 14]. Reduction in the number of EDs over the years seems to have had little or no influence on this picture [6]. Other countries report comparable variation between ED profiles [19–22]. It is notable that with an average volume of about 23,000 patients (range 6000–53,000) and an average of 16 treatment bays (range 4–36) EDs in The Netherlands are relatively small in an international perspective. In 2016, the number of patients seen in an ED equals 116 patients per 1000 inhabitants. For comparison, in Belgium 290/1000, in the UK 264/1000 and in France 279/1000 inhabitants [32]. Together with both a low percentage of self-referrals (average 15%, range 1–56%) and a high percentage hospitalization of patients seen in the ED (average 38%, range 13–76%), in general EDs in The Netherlands might be considered as fairly efficient. At the same time, there is still a broad range of variation among EDs that is insufficiently understood. More insight into backgrounds of this variation and critical interpretation hereof might enable targeted interventions to achieve even more efficiency.

The second aim was to investigate relationships between a selection of volume and resource characteristics on a national level. Because there is no national ED framework yet, we hypothesized relationships as a first method of explorative research. No generally accepted relevant correlation hypotheses are available in literature. Therefore, we had to formulate them ourselves first. Despite limitations, the use of hypotheses can be seen as useful first indicators towards better understanding how available EDs relate to one another in regard to specific characteristics. We formulated six hypotheses around ED patient volumes and hospitalization of ED patients on one side and available resources on the other side and found both positive and significant correlations. Although this might seem obvious at first sight, these findings highlight at the same time the question that explains the differences present. How can it be that EDs with an equal volume of ED patients or hospitalizations do so with huge differences in terms of resources? These and other differences need to be investigated and explained in order to explore where there is actually room to improve quality and reduce costs.

In daily practice, our findings establish a baseline understanding of ED characteristics and mutual relationships. First of all, this can serve as a starter towards the development of an ED resource allocation framework and in line with this as input for an ED benchmark-instrument. This development however does require more comprehensive insight into characteristics of individual EDs, how these departments relate to one another and especially adequate explanations for mutual differences. For example, to compare ED resource planning, information such as distribution of patient volume and admission volume during the day/week/months, severity of the illnesses or injuries treated, and length of stay in the ED together with insight into reasons for admission should be taken into account. Our findings must be seen as a first step and give rise to a more extensive assessment of ED characteristics and interpretation of variation. Secondly, when coupled with mandatory reporting, annual assessment may become an instrument to determine targeted interventions for ED landscape optimization policy for reasons such as cost reduction, quality improvement and preventing crowding on one side and to monitor the effects of this policy in more detail on the other side.

Our study may also give direction to future research aiming to provide additional insight in the ED landscape and the effect of interventions on costs, quality and crowding. Thus far, we have used annual surveys on a voluntary basis. Further studies could benefit from a mandatory national ED registration coupled with more extensive reporting, including variables to correct for case mix differences on the level of patients (e.g., severity/complexity of patients) and ED units themselves (type of hospital, structure with GPC, geographical location). Future research is needed to achieve more and better insight into characteristics of individual EDs, the mutual connection of this, how individual EDs relate to national outcomes and adequate explanations for deviations. Studies are also needed to explore how, as far as ED care is concerned, economies of scale relate to scale disadvantages. In addition, it would be useful to identify objective national volume to resource ratios which can serve as a reference point for local interpretation. Our findings can also be of interest to the international reader. After all, a more detailed insight and understanding of the Dutch ED landscape makes it possible to compare more accurately with the ED landscape in their own country, for example when looking for optimization options.

Although our study has its strengths, like 100% participation and high degree of completeness of requested data, we acknowledge that several limitations may impact study results. The study was based on data obtained from registrations of individual EDs. We were not able to check for accuracy of all registries ourselves. Second, an (inter)nationally validated instrument to characterize EDs does not exist. In this explorative study, we investigated a selection of administrative characteristics. Although among others based on previously conducted and comparable research, we do not pretend to be complete or have used the only best method. Thirdly, as far as we have been able to verify, this is the first study using hypothesis to explore relations between characteristics within the ED landscape. No generally accepted relevant correlation hypotheses are available in literature. Therefore, we had to formulate them first. Although this is not validated, we believe this is a method worthwhile in order to obtain better insight. Despite the limitations, the use of hypotheses can be seen as useful first indicators towards better understanding how available EDs relate to one another in regard to their characteristics. Fourthly, we have studied centrally organized EDs that were operational and available 24 h a day, 7 days a week. Alternative hospital entrances for urgent care were not part of our study, but may be included in the future. Finally, the authors were aware that ideally comparing ED resource planning should take more basic information then patient volume and hospitalization volume into account. Unfortunately, this information was not available. Despite these limitations, our study provides more insight in the ED landscape of The Netherlands than was available to date.

## Conclusions

In conclusion, our study shows that the ED landscape is still pluriform by numbers and specifications of individual ED locations. At the same time, this study identifies on a national level associations between patient and hospitalization volumes on one side and number of ED treatment bays, ED nurse workforce capacity and available hospital beds on the other side. These findings establish a baseline understanding of how EDs relate to one another and form a first step towards more insight into the ED landscape present and might be useful as input for the development of an ED resource allocation framework and more targeted optimization policy in the future.
